# Virus transcript levels and cell growth rates after naturally occurring HPV16 integration events in basal cervical keratinocytes

**DOI:** 10.1002/path.4358

**Published:** 2014-05-21

**Authors:** Cinzia G Scarpini, Ian J Groves, Mark R Pett, Dawn Ward, Nicholas Coleman

**Affiliations:** Department of Pathology, University of CambridgeUK

**Keywords:** human papillomavirus, E6/E7, selection, integration

## Abstract

Cervical carcinogenesis is characterized by a clonal selection process in which the high-risk human papillomavirus (HRHPV) genome usually changes from the extra-chromosomal (episomal) state seen in productive infections to DNA that is integrated into host chromosomes. However, it is not clear whether all HRHPV integration events provide cells with a selective growth advantage compared with the episome-containing cells from which they originate. It is also unclear whether selection of cells containing a particular integrant from a mixed population simply reflects the highest levels of virus oncogene expression or has additional determinants. These early events in cervical carcinogenesis cannot readily be addressed by cross-sectional studies of clinical samples. We used the W12 model system to generate a panel of cervical squamous cell clones that were derived from an identical background under non-competitive conditions and differed only by the genomic site of HPV16 integration. Compared with the ‘baseline’ episome-containing cells from which they were isolated, only 9/17 clones (53%) showed significantly greater growth rates and only 7/17 (41%) showed significantly greater expression of the major virus oncogenes E7/E6. There were significant variations in levels of HPV16 transcription per DNA template, changes that were associated with histone modifications in the integrated virus chromatin. Cell growth rates showed only weak and non-significant associations with protein and mRNA levels for E7, E6, and the mean E7/E6 values. We conclude that HPV16 integration in basal cervical cells does not necessarily lead to increased levels of virus oncogenes, or to a competitive growth advantage, when compared with the initiating episome-containing cells.

## Introduction

There are ∼500 000 new cases of cervical carcinoma per annum worldwide [1]. The large majority are squamous cell carcinomas (SCCs), which arise from precursor squamous intraepithelial lesions (SILs) through a clonal selection process, characterized by the emergence of cells with the greatest competitive growth advantage [2–4]. Whereas low-grade SILs (LSILs) represent non-neoplastic productive virus infections [5], high-grade SILs (HSILs) are abortive infections characterized by clonal expansion of morphologically atypical cells showing elevated levels of virus oncoproteins [6–8].

Infection with high-risk human papillomavirus (HRHPV) is a necessary cause of cervical carcinoma [9,10]. Whereas in productive lesions the virus DNA is maintained in the extra-chromosomal (episomal) state, at ∼100 copies per cell [11,12], in ∼85% of SCCs HRHPV DNA is integrated into the host chromosomes [2,4]. The integrant-containing carcinoma cells show increased expression of the HRHPV oncogenes E6 and E7 [13,14], typically associated with silencing of the virus transcriptional regulator E2 through deletion, truncation or epigenetic silencing [14–16]. In addition, selection of cells containing integrated HRHPV DNA requires loss of residual virus episomes, which produce E2 capable of repressing integrated virus DNA in *trans* [4,17,18]. In *in vitro* models of cervical squamous carcinogenesis, the integrant-containing cells that emerge from mixed populations of episome-containing cells have a selective growth advantage [3,18].

An alternative route of cervical carcinogenesis is characterized by episome retention and shows similarities to integrant-associated progression [19]. In particular, there is selection of cells with deregulation of episome-derived transcription (compared with the episome-containing cells of productive virus infections), leading to elevated virus oncogene expression levels and a competitive growth advantage [19].

Several important questions concerning the biology of HRHPV integration and cervical carcinogenesis remain poorly addressed. First, do all integration events (when derepressed following episome loss) lead to increased levels of virus oncogenes and/or a selective growth advantage, compared with the episome-containing cells from which they originated? Second, does selection of a particular integrant simply reflect the greatest levels of virus oncogene expression per cell, or are there additional determinants? These questions cannot be answered by cross-sectional analysis of cervical neoplasms, which by definition contain integrants with the greatest competitive advantage. Moreover, clinical samples do not allow longitudinal investigations of events preceding integrant selection and may be confounded by the effects of epithelial differentiation on the HRHPV life cycle and gene expression. Of the available experimental systems for longitudinal studies of early events in cervical carcinogenesis, the most useful to date has been the W12 model [17,20,21].

Parental W12 cells represent a polyclonal population of cervical keratinocytes (squamous epithelial cells) generated following primary culture of a productive lesion (cervical LSIL) that arose following natural infection with HPV16, the most common HRHPV type in cervical SCC. At early passages of W12, HPV16 is able to persist stably at ∼100–200 episomal copies per cell [11]. We have used continuous *in vitro* passage to generate multiple long-term culture series of W12 cells [19,22]. In these, there is usually breakdown of episome persistence, associated with the emergence of cells containing integrated HPV16. These events are associated with chromosomal instability, acquisition of genomic copy number imbalances, and phenotypic progression from LSIL through high-grade SIL (HSIL) to SCC. All of these *in vitro* changes closely mirror cervical neoplastic progression *in vivo* [19,22].

We have previously used the W12 model to characterize the range of integration events that occur following naturally acquired HPV16 infection, prior to episome loss and integrant selection [21]. For this, we used W12 long-term culture series-2 (W12Ser2), in which an integrant at 8q24.21 emerged after approximately 24 passages [where one passage (p) represents ∼6 cell doublings] [18]. We studied populations of W12Ser2 cells from early time-points prior to the emergence of the 8q24.21 integrant (ie p12 or p13), when only episomes were detectable by Southern blot and the cells reformed an LSIL in organotypic tissue culture [19,21]. We used single cell cloning under non-competitive conditions, in which episome-derived E2 was present (thereby providing a repressive environment for integrants) until after the cloning process. While several of the clones that were isolated showed integration at 8q24.21, many others showed integration elsewhere in the genome. In total, we isolated 24 clones that each contained a different HPV16 integration site [21]. Interestingly, despite the cells being isolated in a non-competitive environment, the range of integration sites seen overlapped closely with those observed in cervical SCC *in vivo* (ie following a clonal selection process) [2,4,23,24]. This observation argued that HPV16 integrates at sites in the human genome that are relatively accessible for insertion of foreign DNA [25,26].

>The clones isolated from W12Ser2 cells represent a unique resource, as they were derived from an identical cellular background and differ only by the site of HPV16 integration. In the present study, we performed a detailed investigation of cell phenotype and virus early gene expression levels across the clones, with reference to (i) normal cervical keratinocytes (NCx); (ii) the ‘initiating’ episome-containing W12Ser2 cells from which the clones were generated, which reformed an LSIL in organotypic tissue culture [18,21]; and (iii) cells of the integrant clone that spontaneously emerged during long-term culture of W12Ser2. The latter had been cultured continuously to p31 and were referred to as W12Ser2p31 cells. This *in vitro* approach has provided insights into the mechanism of selection of cells containing integrated HRHPV that cannot readily be obtained using clinical samples or animal models.

## Materials and methods

### Cell culture and nomenclature

The W12 cell line system has been described in detail previously [11,18,19,22]. Cells were routinely authenticated by detection of HPV16 DNA and by identification of characteristic genomic copy number imbalances [20,22]. Long-term culture of polyclonal W12Ser2 has been described previously [18,19,21]. By Southern blotting, only episomes were detectable to p18, followed by spontaneous episome clearance and the selection of cells with HPV16 integrated at 8q24.21. Only integrated HPV16 DNA was detectable from p24 [18].

The integrant-containing W12 clones were generated under non-competitive conditions from W12Ser2 p12 or p13 cells, which reformed LSIL epithelia in organotypic tissue culture [18,19,21]. At these early passages, the W12Ser2 cells stably maintained episomes and expressed E2 that was able to silence integrant-derived transcription [18]. Consequently, there were no competitive pressures to favour selection of any particular integrant. For each clone, the site of integration into the host DNA was determined from genomic DNA using restriction site-PCR or from RNA using rapid amplification of cDNA ends (RACE)-PCR [21,23,27].

In the present study, we used all of the clones available for *in vitro* characterization. These numbered 17 in total, representing 16 of the clones described previously [21] plus an additional clone (clone J3) with integration at 8q24.21. None of the clones showed evidence of residual episomes on Southern blotting. Details of each clone, including the sites of HPV16 integration, are given in Supplementary Table 1 and our previous publication [21]. Importantly, the integrant-containing cells were studied at the lowest available passage after cloning (generally between p3 and p8), in order to minimize any effects of genomic instability caused by deregulated HPV16 oncogene expression [28].

We compared our findings in the integrant-containing clones with those in the ‘initiating’ episome-containing W12Ser2 cells from p10 to p12, as well as the spontaneously selected W12Ser2 cells at p31 (W12Ser2p31). We also used NCx/6 cells, derived from primary cultures of normal uninfected ectocervical keratinocytes obtained from a hysterectomy specimen performed for disease unrelated to the cervix [19].

All cells were grown in monolayer culture using irradiated 3T3J2 fibroblast feeder cells and serum/growth factor-containing medium, as previously described [29]. Such monolayer cultures were used to restrict cell differentiation and maintain the phenotype of the basal epithelial cell layer, the key site of HRHPV transcriptional deregulation in cervical carcinogenesis [4,30]. All feeder cells were removed before the keratinocytes were harvested. Cell doubling times and phenotype in organotypic tissue culture were determined as previously described [22,29]. Further details are given in the Supplementary materials and methods.

### Quantification of HPV16 gene copy number, transcript levels, and protein abundance

Copy numbers of the HPV16 early genes E7, E6, and E2 were determined using SYBR Green (Sigma-Aldrich, St Louis, MO, USA) quantitative PCR (qPCR) of genomic DNA, as described elsewhere [17] (Supplementary Table 2). Transcript levels per cell of the HPV16 early genes E7, E6, and E2 were determined using SYBR Green quantitative reverse transcription-PCR (qRT-PCR) (Supplementary Table 3). In this, the E7 primers detected all transcripts encoding the E7 protein. We used a range of primer pairs to quantify total E6 transcripts, as well as the alternatively spliced forms, E6*I, E6*II, E6*III, E6*IV, E6*X, and full length E6 [31]. Separate primer pairs were used to quantify the 5′ and 3′ ends of E2 [32]. In addition, we combined the HPV16 expression and gene copy number quantification data to determine the levels of transcription per DNA template copy for E7, E6, and E2, as previously described [19]. Levels of HPV16 proteins were measured using quantitative western immunoblotting, as previously described [17,19]. Full details of qPCR and quantitative immunoblotting may be found in the Supplementary materials and methods.

### Chromatin immunoprecipitation (ChIP)

ChIP analysis of histone modifications was performed as previously described [19]. PCR quantification of HPV16 DNA sequences in the immunoprecipitated chromatin covered six sites along the HPV16 genome, from the long control region to the E1 open reading frame (Supplementary Table 4). Further details are given in the Supplementary materials and methods.

## Results

### Cell growth and epithelial morphology

**Figure 1:**
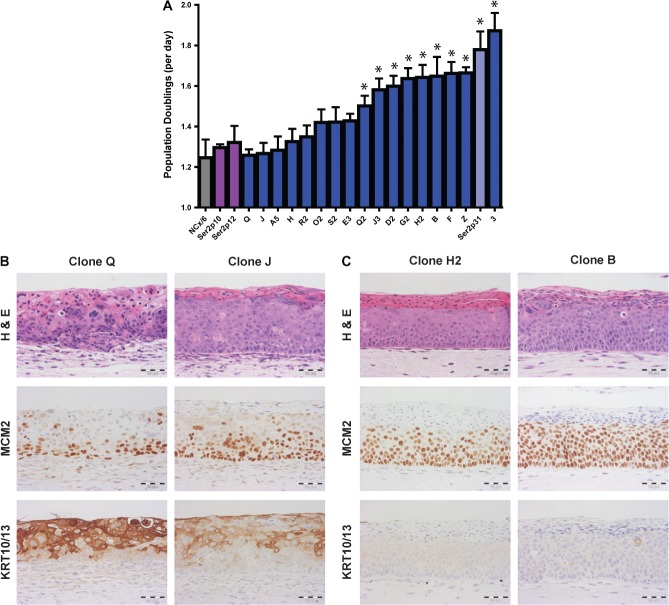
Cell growth in monolayer culture and phenotype in organotypic tissue culture. (A) Population doublings per day in W12 clones (blue bars), the spontaneously selected W12Ser2p31 cells (pale blue bar), the initiating episome-containing W12Ser2p10 and W12Ser2p12 cells (purple bars), and NCx/6 (grey bar). Error bars = SEM. An asterisk denotes cells with significantly faster growth than the mean of the W12Ser2p10 and W12Ser2p12 cells (*p* < 0.01). (B, C) Appearances of the epithelia reformed in organotypic culture by representative W12 clones, for which growth rate in monolayer culture was low (Q and J) or high (H2 and B). Tissue sections were stained by H&E and immunohistochemistry for the cell-cycle marker MCM2 and the squamous cell differentiation markers KRT10/13. Scale bars = 50 µm.

Across the 17 W12 clones available for analysis, the number of population doublings per day in monolayer culture varied by ∼1.5-fold (Figure 1A), equating to an ∼17-fold difference in the number of population doublings if extended over 1 week. Predictably, the cells that had been spontaneously selected during long-term culture of W12Ser2 (W12Ser2p31) had one of the highest population doubling rates, with none of the clones showing a significantly greater rate. The number of population doublings per day of the initiating episome-containing W12Ser2 cells was ∼75% of that of the W12Ser2p31 cells and not significantly greater than that of NCx/6 cells (Figure 1A). Interestingly, only 9/17 clones (53%) showed a significantly greater population doubling rate than the initiating episome-containing W12Ser2 cells (*p* < 0.01).

We used three-dimensional organotypic tissue culture to test whether the differences in cell growth rates across the clones in monolayer culture were reflected in differences in the morphology of the epithelia reformed by the cells *in vitro*. For two representative clones (Q and J) (Figure 1B) in which cell growth was no greater than that of the initiating episome-containing cells, only the basal ∼1/3 of the reformed epithelium contained morphologically atypical cells that were in cell cycle (as evidenced by expression of MCM2). Cells in the upper ∼2/3 of the epithelium showed evidence of differentiation on H&E staining, associated with expression of the squamous epithelial differentiation markers KRT10/13. Together, these histological changes resembled LSIL. In contrast, for two representative clones (H2 and B) (Figure 1C) in which cell growth was significantly greater than that of the initiating episome-containing cells (*p* < 0.01), atypical cycling cells extended into the upper ∼1/3 of the epithelium. This was associated with little evidence of differentiation on H&E staining, and with an almost complete absence of KRT10/13 expression, save for occasional individual dyskeratotic cells. Together, these changes were of higher grade than the epithelia reformed by clones Q and J and resembled HSIL. None of the clones examined showed evidence of invasion in organotypic tissue culture. We concluded that comparative measurements of cell growth in monolayer culture were valid indicators of cell phenotype (proliferation and differentiation) in stratified epithelia.

### HPV16 transcript levels per cell

**Figure 2:**
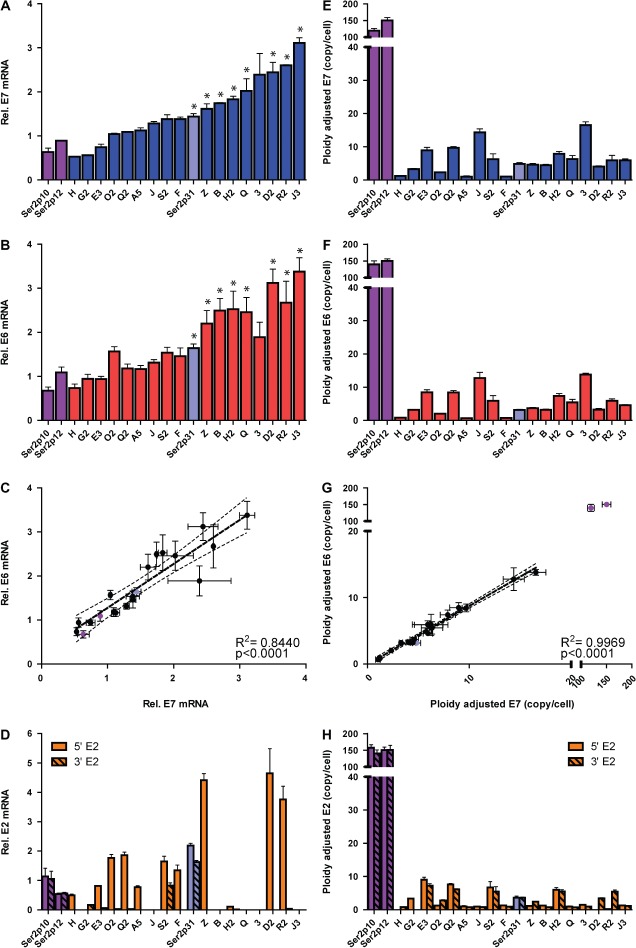
Quantification of virus gene expression and copy number. The charts show levels of expression (left column) and copy number (right column) for HPV16 E7 (panels A, E), total E6 (panels B, F), and E2-5'/E2-3' (panels D, H). Expression levels were referenced to those in W12Ser6p11. Relationships between the E7 and E6 levels are shown in panels C and G, including correlation data for the 17 clones. The pale blue bars/circles show data for the spontaneously selected W12Ser2p31 cells, while the purple bars/circles show data for the initiating episome-containing W12Ser2p10 and W12Ser2p12 cells. Data for the 17 clones are colour-coded by gene (E7: blue bars; E6: red bars; E2: orange bars). In each bar chart, the clones are ordered by increasing levels of E7 transcript per cell (ie the order determined in the analysis shown in panel A). Rel = relative. Error bars = SEM. An asterisk denotes cells where E7 or E6 values were significantly higher than the mean of the W12Ser2p10 and W12Ser2p12 cells (*p* < 0.01).

Across the 17 clones, levels per cell of HPV16 E7 transcripts varied by ∼6-fold (Figure 2A) and levels of HPV16 total E6 transcripts by ∼5-fold (Figure 2B). When compared with the initiating episome-containing W12Ser2 cells, only 7/17 (41%) showed significantly higher levels of E7 (*p* < 0.01) and only 7/17 (41%) clones showed significantly higher levels of E6 (*p* < 0.01). Interestingly, in W12Ser2p31 cells, which grew rapidly in monolayer culture and were spontaneously selected from the polyclonal W12Ser2 cultures [18], levels of E7 and E6 were relatively moderate (Figures 2A and 2B), with two clones (R2 and J3) showing significantly higher levels of E7 and E6 (*p* < 0.01). Across the 17 clones, there was a highly significant correlation between the levels of E7 and total E6 transcripts per cell (*p* < 0.0001) (Figure 2C). In addition, levels of E7 and total E6 correlated with levels of the alternative E6 transcripts E6*I, E6*II, and E6*X, as well as E6 full length (Supplementary Figure 1).

We quantified levels of the 5′ and 3′ ends of the E2 transcript separately, given our previous finding that the E2 gene is typically disrupted in the integrant-containing clones [4,21], reflecting a common feature of the HRHPV integrants seen in cervical SCC *in vivo* [14,16]. Of the 17 clones, five (29%) showed loss of all E2 transcripts, while only one (S2) showed substantial levels of E2-3′ transcripts (Figure 2D). Eleven clones expressed E2-5′ transcripts, with the levels varying by ∼44-fold. There was no overall correlation between levels of E2-5′ transcripts and those of E7, total E6 or any alternative E6 transcript (data not shown). The selected W12Ser2p31 cells expressed both E2-5'and E2-3′, similar to clone S2.

### Relationship between HPV16 transcript levels and gene copy number

We tested whether the wide range in gene expression levels could be explained by differences in HPV16 DNA template abundance. We used qPCR to quantify gene copy number for HPV16 E7, E6, E2-5′, and E2-3′ (Supplementary Table 2). The E7 and E6 copy numbers ranged from ∼1 to 14 and from ∼1 to 16, respectively (Figures 2E and 2F), with a highly significant correlation between the values (*p* < 0.0001) (Figure 2G). With the exception of four clones (E3, Q2, S2, and H2), copy numbers of E2-5′ and/or E2-3′ were 1 or 0 (Figure 2H) (Supplementary Figure 2). This supported previous Southern blot evidence [21] and indicated the absence of full-length virus concatemerization (ie the clones harboured so-called type I integrants) [13]. Some of these clones nevertheless contained multiple copies of E6/E7, consistent with effects of local DNA rearrangements following integration [33–35]. Across all the clones, there was no correlation between transcript levels and gene copy number for E7, total E6 or E2-5' (Supplementary Figures 3A–3C).

**Figure 3:**
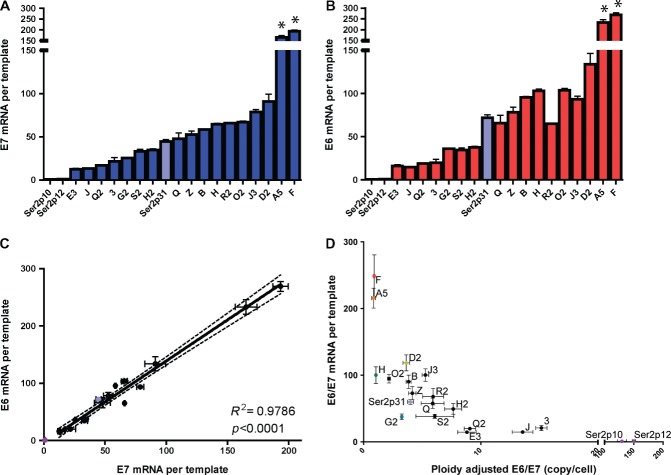
Levels of HPV16 gene expression per template. Values are shown for E7 (A) and E6 (B), with correlations between the values for the 17 clones shown in C. The pale blue bars/circles show data for the spontaneously selected W12Ser2p31 cells, while the purple bars/circles show data for the initiating episome-containing W12Ser2p10 and W12Ser2p12 cells. The bars for the 17 clones are colour-coded by gene (E7: blue bars; E6: red bars). In each bar chart, the clones are ordered by increasing levels of E7 expression per template (ie the order determined in the analysis shown in panel A). Error bars = SEM. An asterisk denotes cells where values were significantly higher than the W12Ser2p31 cells (*p* < 0.01). Panel D shows a plot of mean E6/E7 expression per template versus template copy number per cell. While individual W12 clones are generally represented by black circles, specific colours are used to highlight the clones used for ChIP–qPCR.

The range of transcript levels per template varied for both E7 and total E6 by ∼16-fold and ∼17-fold, respectively (Figures 3A and 3B). Interestingly, expression levels per template for the spontaneously selected W12Ser2p31 cells were relatively moderate, with 2/17 (12%) clones (A5 and F) showing significantly greater levels of E7 and E6 expression per template (*p* < 0.01). Across all clones, there was a highly significant correlation between levels of E6 and E7 expression per template (*p* < 0.0001) (Figure 3C). Transcript levels per template for E2-5′ (where expressed) varied by ∼13-fold (Supplementary Figure 4), with no correlation with transcript levels per template for E7, total E6 or E2-3′ (data not shown).

Given the close correlation between levels of E6 and E7 expression per template, the values were combined to produce a mean level of E6/E7 mRNA expression per template. When this parameter was plotted against mean E6/E7 DNA copy number, we observed an overall negative relationship, with high DNA copy number clones showing relatively low levels of expression per template and vice versa (Figure 3D). However, there were multiple examples where clones that contained the same or very similar DNA copy number (with no evidence of full-length virus concatemerization) showed significant differences in levels of E6/E7 expression per template. For example, of the clones with three or four E6/E7 copies per cell, clone D2 showed ∼3.7-fold greater expression per template than clone G2 (*p* = 0.036), while of the clones with one copy per cell, clone F showed ∼3.3-fold greater expression per template than clone H (*p* = 0.007).

### Relationship between transcript levels per template and virus chromatin modifications

**Figure 4:**
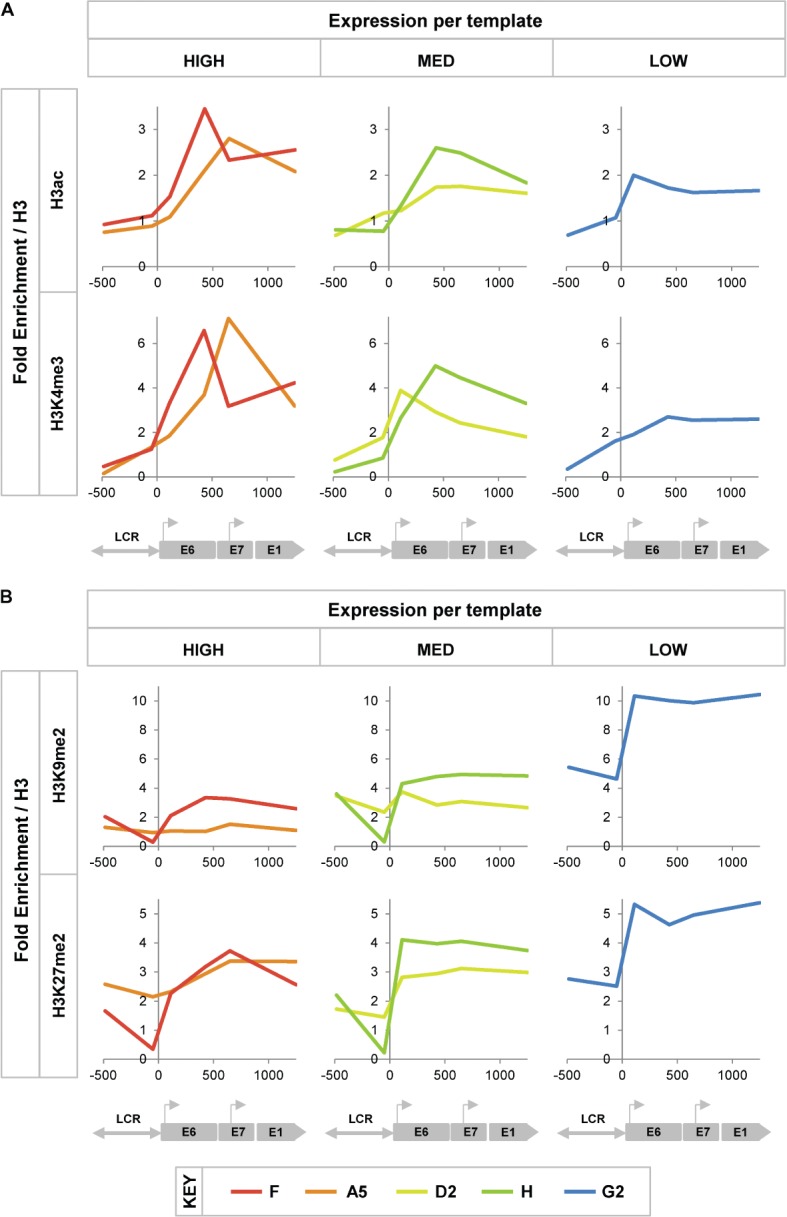
Histone modifications in integrated HPV16 chromatin. In each graph, the *y*-axis shows fold enrichment of histone H3 modifications, referenced to background H3 levels. The *x*-axis and underlying schematic show the region of the HPV16 genome analysed by qPCR (LCR = long control region). Histone modifications of active chromatin are shown in panel A and modifications of repressed chromatin in panel B. The columns show data for clones in which transcription levels per template were high (left), medium (MED; middle) or low (right).

We hypothesized that the significant variations in E6/E7 expression per template were due to epigenetic differences in the virus chromatin related to transcriptional control. We therefore used ChIP–qPCR to measure levels of histone modifications associated with transcriptionally active or repressed DNA templates. To avoid any complexity caused by heterogeneous changes in cells containing concatemerized integrants, we focused on clones where E6/E7 copy number was 4 or less and there was no retention (or expression) of either E2-5′ or E2-3′. From this group, we selected two clones with high levels of expression per template (F and A5), two with medium levels (D2 and H), and the one available clone with low levels (G2). Across these clones, increasing levels of virus expression per template were associated with increasing levels of histone marks of transcriptionally active DNA templates (acetylated histone H3 and trimethylated lysine 4 of histone H3) (Figure 4A) and with decreasing levels of histone marks of transcriptionally repressed DNA templates (dimethylated lysine 9 of histone H3 and dimethylated lysine 27 of histone H3) (Figure 4B).

### HPV16 protein levels and cell growth rates

**Figure 5:**
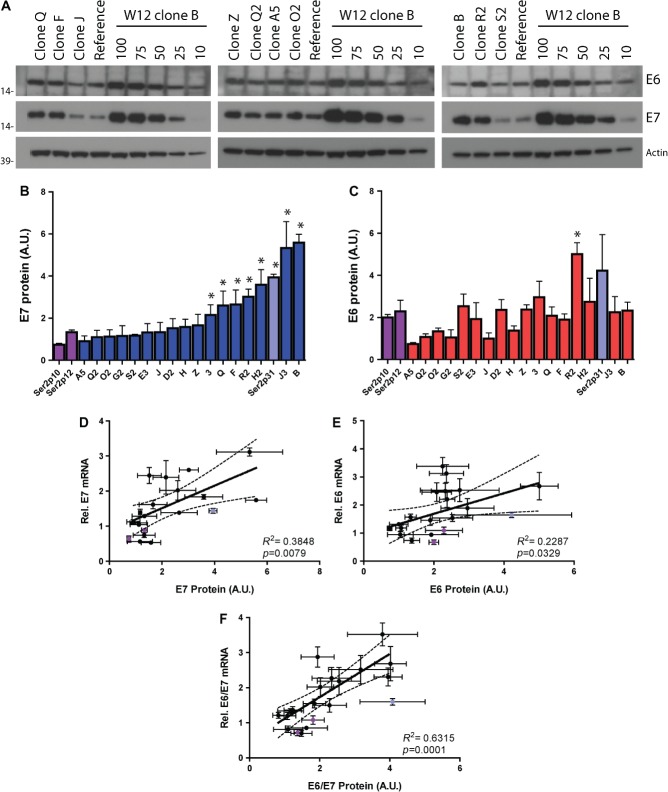
Expression levels of HPV16 early proteins per cell. Panel A shows western blots for HPV16 E6 and E7 protein expression in representative W12 clones. The reference samples were from independent episome-containing W12Ser6p11 cells. The right-hand five lanes in each blot show the serial dilution of W12 clone B used to generate the standard curve for protein quantification, with the numbers denoting the µg amounts of total protein per lane. The bar charts show protein levels for E7 (B) and E6 (C). The pale blue bars show data for the spontaneously selected W12Ser2p31 cells, while the purple bars show data for the initiating episome-containing W12Ser2p10 and W12Ser2p12 cells. An asterisk denotes cells where values were significantly higher than the mean of the W12Ser2p10 and W12Ser2p12 cells (*p* < 0.01). Relationships between protein and transcript levels are shown for E7 (D), total E6 (E), and the mean E7/E6 values (F), including correlation data for the 17 clones (black circles). In all panels, error bars = SEM. A.U. = arbitrary units.

Across the 17 clones, levels of HPV16 E7 and E6 proteins per cell each varied ∼6-fold (Figures 5A–5C). When compared with the initiating episome-containing W12Ser2 cells, only 7/17 (41%) clones showed significantly higher levels of E7 (*p* < 0.01), while only 1/17 (6%) showed significantly higher levels of E6 (*p* < 0.01). Full-length E2 protein was not detectable in any clone (Supplementary Figure 5B), consistent with the general absence of E2-3′ transcripts (Figure 2D). Across the clones, there was a significant correlation between levels of mRNA and protein for E7 (*p* = 0.008) (Figure 5D), total E6 (*p* = 0.03) (Figure 5E), and the mean E6/E7 values (*p* = 0.0001) (Figure 5F). Levels of E6 protein did not correlate with any of the alternative E6 transcripts individually (Supplementary Figures 6A–6 F), although E7 protein did correlate significantly with levels of E6*I (*p* = 0.0005) and E6*II transcripts (*p* < 0.0001) (Supplementary Figures 6H and 6I).

**Figure 6:**
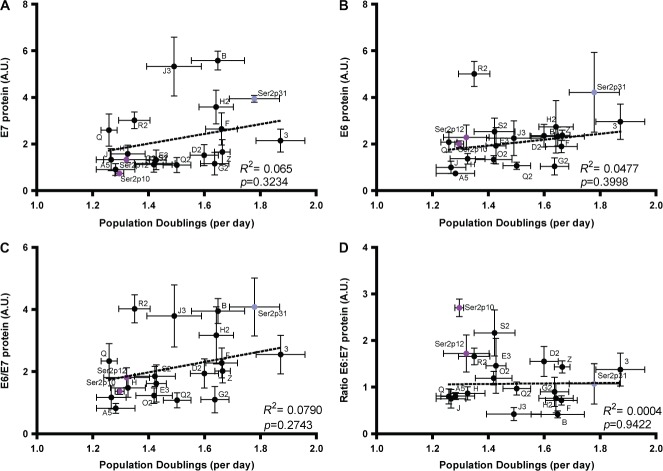
Relationships between cell proliferation rates and HPV16 oncoprotein levels. The graphs plot cell growth rate versus protein levels for E7 (A), E6 (B), the mean E6/E7 values (C), and the E6:E7 ratios (D). The correlation data are for the 17 clones (black circles). Results for the W12Ser2p31 cells (pale blue circles) and W12Ser2p10 and W12Ser2p12 cells (purple circles) are also shown. In all panels, error bars = SEM. A.U.; arbitary units.

Cell growth rates showed only weak and non-significant associations with protein levels of E7 (Figure 6A), E6 (Figure 6B), and the mean E6/E7 values (Figure 6C). There was no association between E6:E7 protein ratios and cell growth rates (Figure 6D). There were multiple instances where cells with similar E6/E7 protein levels showed significant differences in growth rates. This applied to clones where levels of E6/E7 proteins per cell were low (eg G2 versus A5, *p* = 0.023) or higher (eg 3 versus Q, *p* = 0.009). These observations were mirrored by evidence of only weak and non-significant associations between growth rate and transcript abundance for E7 (Supplementary Figure 7A), total E6 (Supplementary Figure 7B), and the mean E6/E7 values (Supplementary Figure 7C). Likewise, there were only weak and non-significant correlations between growth rates and levels of the alternative E6 transcripts, both individually (Supplementary Figures 8A–8 F) and when combined with E7 to produce mean values (Supplementary Figures 8G–8L). There was no association between total E6:E7 transcript ratios and cell growth rates (Supplementary Figure 7D).

## Discussion

Basal cells derived from non-neoplastic productive HRHPV infections typically contain ∼100 episomes per cell [11,12]. As integration generally involves a small proportion of these episomes, the residual episomes must be cleared from cells in order to remove E2 that is capable of repressing integrant-derived transcription in *trans* [18]. The W12 clones studied here were derived from W12 culture series-2 cells in which only episomes were detectable by Southern blot and expression from integrants was repressed [21]. The clones therefore contained naturally occurring HPV16 integration sites, regardless of their selectability in mixed cell populations.

This panel of clones has demonstrated that integration of HPV16 in basal cervical squamous epithelial cells does not necessarily lead to increased levels of virus oncogene expression, or to a competitive growth advantage, when compared with the initiating episome-containing cells that reformed an LSIL in organotypic tissue culture. Indeed, only 53% of the integrant-containing clones had a growth advantage compared with the episome-containing cells from which they were derived. This finding strongly supports our experimental strategy of using single cell cloning under non-competitive conditions to isolate cells containing individual HPV16 integrants [21], as the eight clones without a relative growth advantage would not have outgrown the parental episome-containing cells and would not have emerged from the mixed cell population while episome-containing cells remained. We consider that the differences in growth rates observed in monolayer culture were valid indications of cell phenotype, as they correlated with the morphology of the epithelia reformed by the clones in organotypic tissue culture. This finding is consistent with previous observations in studies of episome-associated cervical carcinogenesis, where increased cell growth rates (associated with deregulated episome-derived transcription) were mirrored by phenotypic progression in organotypic tissue culture, from LSIL through HSIL to SCC [19,36].

We deliberately studied the W12 clones at the earliest available passage, in order to minimize any confounding effects of genomic instability caused by deregulated HPV16 gene expression [28]. It is difficult to investigate early events in cervical neoplastic progression by cross-sectional analysis of clinical samples. Carcinoma tissues are not suitable, as progression to malignancy is associated with cell selection, dynamic changes in HRHPV gene expression (whether the cells contain integrants or episomes), and the accumulation of global host genomic imbalances [4,6–8,19]. The available data on HPV expression levels in SILs [37] were mostly derived from HSILs (CIN2/3), in which clonal selection of growth-advantaged cells would have occurred [6,7], plus some LSILs (CIN1) that included the terminally differentiated upper strata where E6/E7 levels increase during the normal HPV life cycle [30,38]. In future work, it will be interesting to determine virus expression levels in microdissected basal epithelial layers of episome-containing productive HRHPV infections (cervical LSILs), which would provide an appropriate reference for studies of integrant-containing cells. In the meantime, in order to establish definitively the effects of HRHPV integration on virus gene expression in the absence of any effects of cell selection or differentiation, it is necessary to compare episome-containing and integrant-containing basal type cells in monolayer culture, as performed in the present study.

Across the 17 W12 clones studied, levels of virus gene expression per cell did not correlate with DNA template abundance. Indeed, expression levels per template varied up to ∼17-fold. For all integrants, expression levels per template were greater than in the initiating episome-containing cells, indicating the different transcriptional environment of integrated versus episomal HPV16 genomes [4,13]. The majority of clones showed no evidence of concatemerization of full-length virus DNA (ie were type I integrants). By studying representative clones, we observed that variations in levels of transcription per template were associated with epigenetic differences in virus chromatin. High-level expression per template was associated with greater abundance of histone marks associated with active chromatin and reduced abundance of marks associated with repressed chromatin [39]. Interestingly, levels of the active chromatin marks were greatest over the transcribed virus exons downstream of the transcription start site, consistent with observations in the HPV18 integrants found in the cervical adenocarcinoma cell line HeLa [40]. This epigenetic variation in integrated HPV16 DNA reflects changes in episome-associated cervical carcinogenesis [19], where deregulation of HPV transcription was associated with changes in histone acetylation in viral episomes, downstream of the transcription start sites.

In future work, it will be important to study the causes and consequences of histone modifications in the W12 clones and whether high-level transcription from integrated HPV16 may be reduced using epigenetic therapies. Where HRHPV DNA is integrated as concatemerized full-length copies (ie as type II integrants) [13], the overall virus chromatin state is likely to be heterogeneous, as transcription sites may be restricted to the 3' virus–host junctions [41]. However, the levels of transcription per DNA template at such junctions may be related to histone modifications similar to those identified in the present study.

The overall correlations that we observed between virus mRNA and protein levels per cell argue against significant post-transcriptional regulation in the integrant-containing W12 clones. While there was some association between growth rates and levels of virus oncoproteins, this was weak and statistically not significant. As the functions of HRHPV E6 and E7 are interconnected [2,4,6–8], it is conceivable that there may an optimal ratio of E6:E7 levels for stimulation of cell proliferation. However, we found no evidence of this in our dataset. There were significant differences between the growth rates of clones showing the same E6/E7 protein levels, at both low and high protein levels per cell. These data argue strongly for a host contribution to the growth of HPV16 integrant-containing basal cervical keratinocytes. It will now be important to investigate the relative contributions of host genes at or near the integration sites, the expression and copy number of which may be directly affected by HPV16 integration [33–35,42], compared with genes elsewhere in the genome, which may be deregulated indirectly, for example through stochastically acquired copy number imbalances and/or mutations [28]. There is evidence of increased genomic instability in integrant-containing cells selected in mixed cell populations [19,22]. Interestingly, HPV oncogenes expressed from episomes may also induce host genomic imbalances that could contribute to viral integration and potentially provide a selective growth advantage [25,26].

Finally, it should be noted that while the panel of integrant-containing cells studied here was generated under non-competitive conditions, regardless of their relative growth advantage, all were isolated in monolayer culture and required retention of the E6 and E7 genes. It is conceivable that other HPV16 integration events occur *in vivo* that disrupt E6/E7 and are therefore ultimately non-selectable. Such integrants would not have been isolated by the strategy that we adopted. They may be identified by high-throughput sequencing analysis of episome-containing productive lesions (LSILs), prior to the selection pressures induced by episome clearance and loss of *trans*-repressive E2.

## Supporting Information On The Internet

The following supporting information may be found in the online version of this article.

Figure S1 Expression of alternative E6 transcripts.

Figure S2 Relationship between DNA copy number for HPV16 E2-5' and E2-3'.

Figure S3 Relationships between expression levels and DNA copy number for HPV16 E7 (A), E6 (B), and E2-5' (C).

Figure S4 Expression levels per template of HPV16 E2-5' and E2-3' mRNA.

Figure S5 Western blot analysis of HPV16 E2 protein levels in representative samples.

Figure S6 Relationships between levels of HPV16 oncoproteins and alternative E6 transcripts.

Figure S7 Relationships between cell growth rates and HPV16 transcript levels.

Figure S8 Relationships between cell growth and alternative E6 transcripts.

Table S1 Summary of the W12 cells studied.

Table S2 Primers and conditions for qPCR of HPV16 and cellular gDNA.

Table S3 Primers and conditions for qRT-PCR of HPV16 and housekeeping transcripts.

Table S4 Primers and conditions for ChIP–qPCR of HPV16 and cellular chromatin.
