# Supplementary figures and images for: Virus transcript levels and cell growth rates after naturally occurring HPV16 integration events in basal cervical keratinocytes
Source: J Pathol. 2014 May 21;233(3):281–93. doi: 10.1002/path.4358 (PMC4285939; doi:10.1002/path.4358)

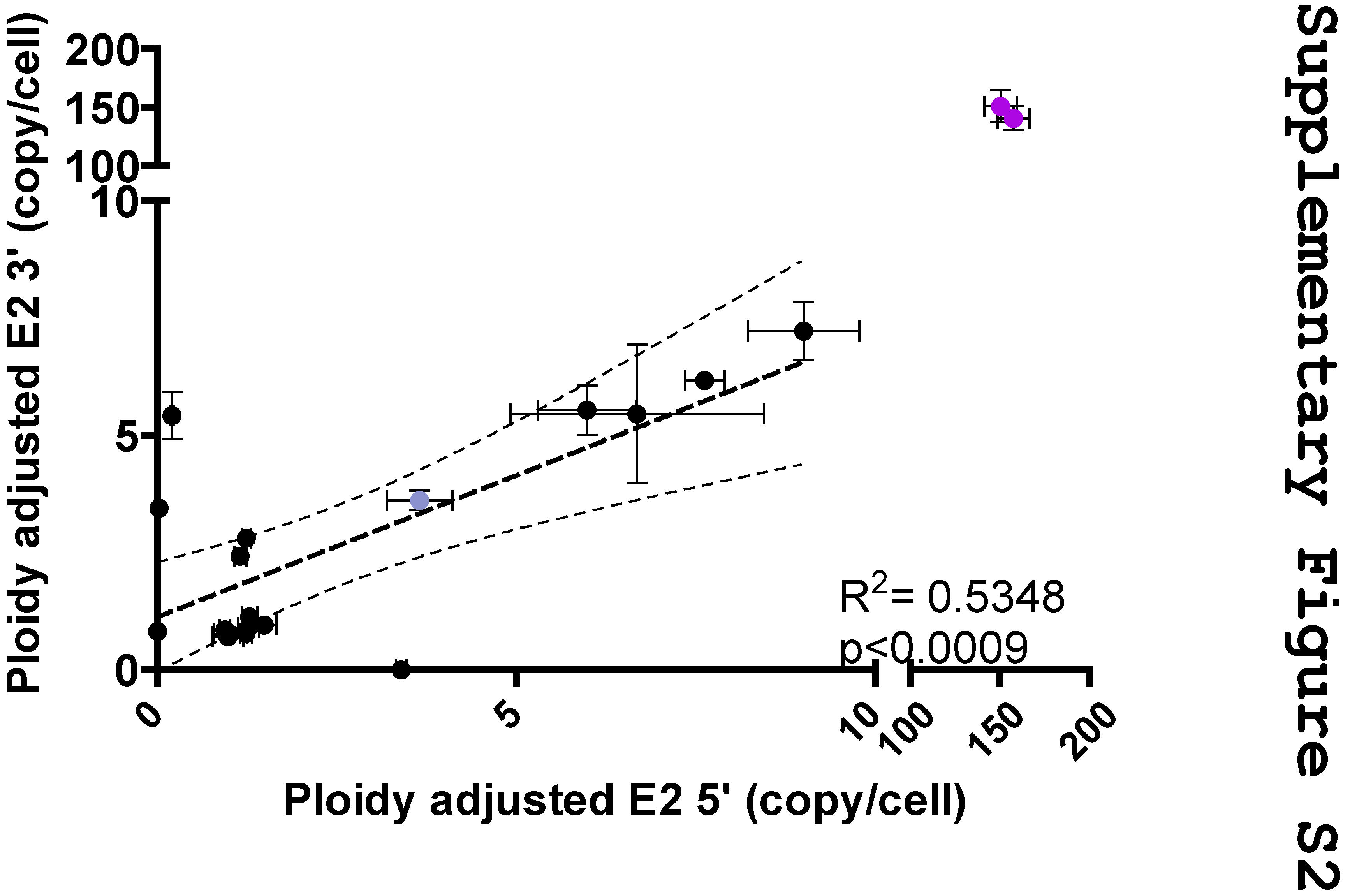

Supplement: Figure S2 — Relationship between DNA copy number for HPV16 E2-5' and E2-3'. [file path0233-0281-SD3.tif]

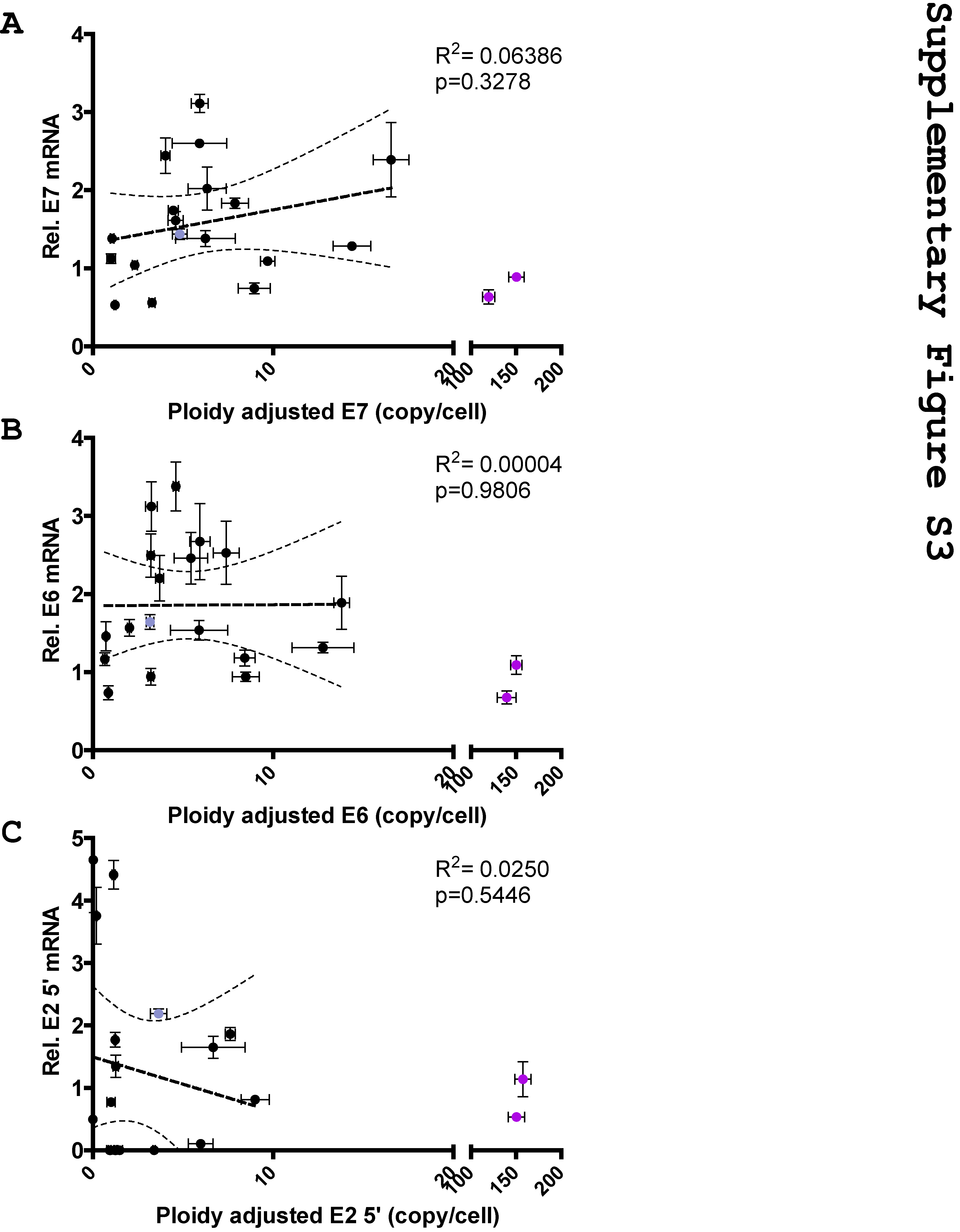

Supplement: Figure S3 — Relationships between expression levels and DNA copy number for HPV16 E7 (A), E6 (B), and E2-5' (C). [file path0233-0281-SD4.tif]

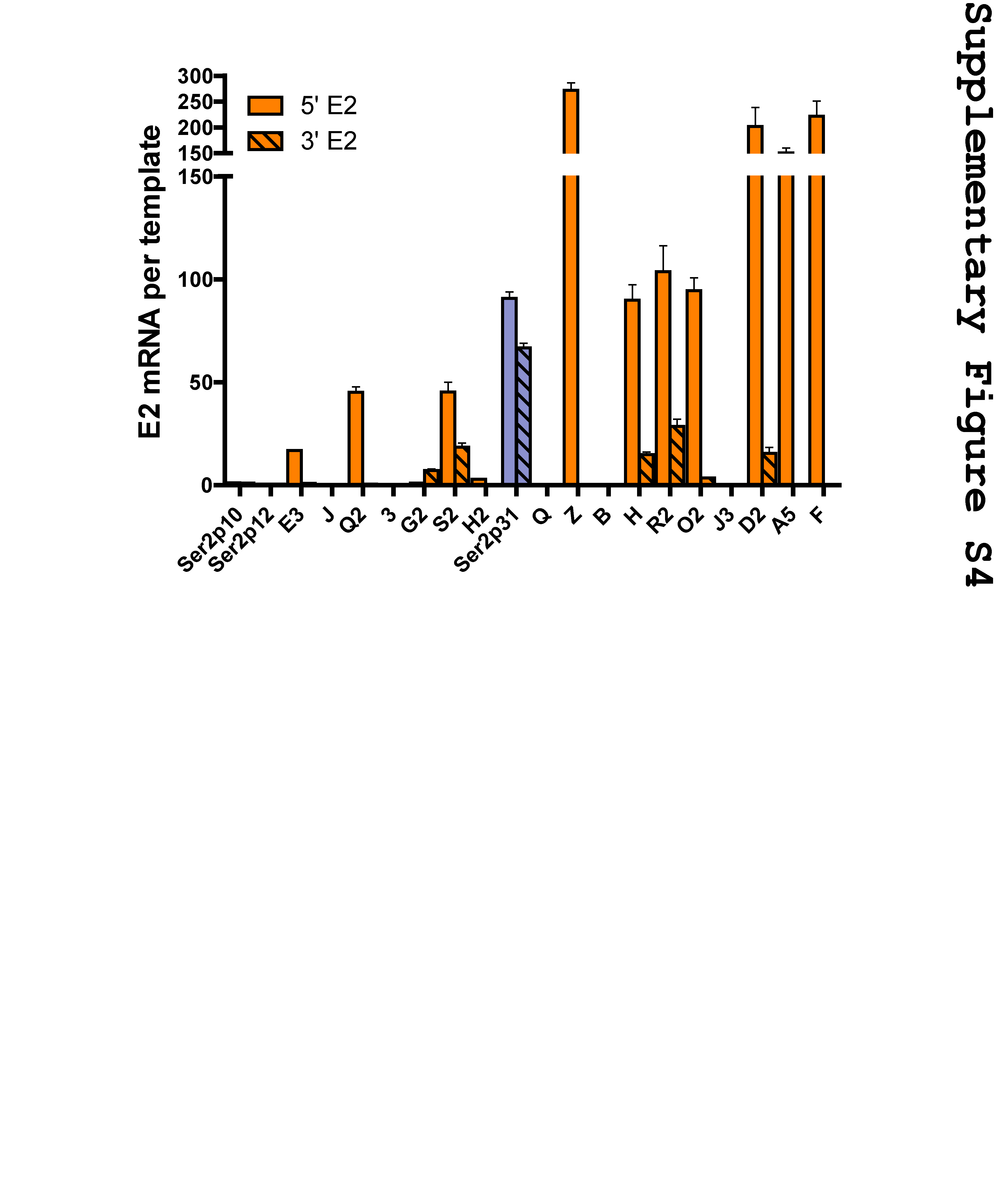

Supplement: Figure S4 — Expression levels per template of HPV16 E2-5' and E2-3' mRNA. [file path0233-0281-SD5.tif]

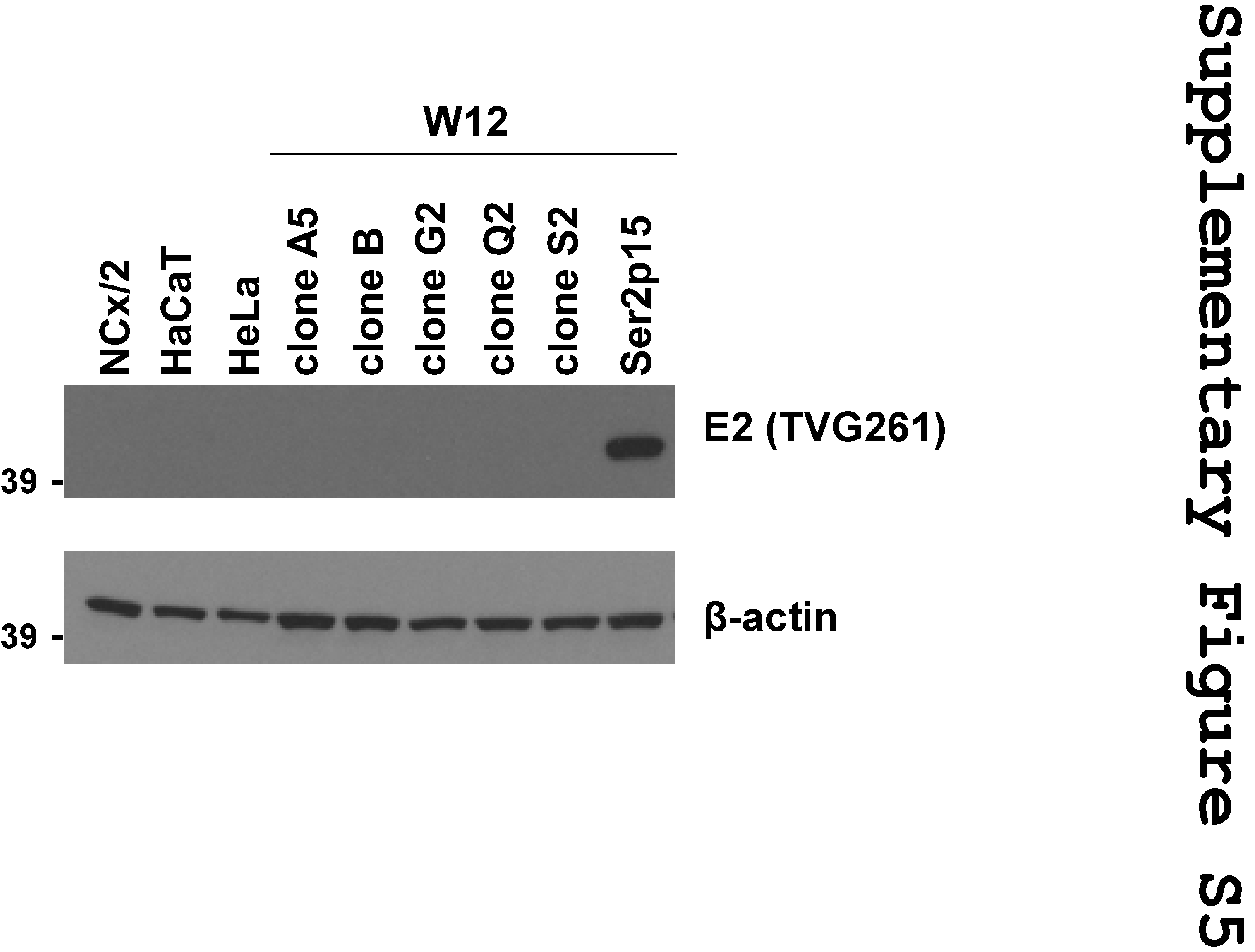

Supplement: Figure S5 — Western blot analysis of HPV16 E2 protein levels in representative samples. [file path0233-0281-SD6.tif]

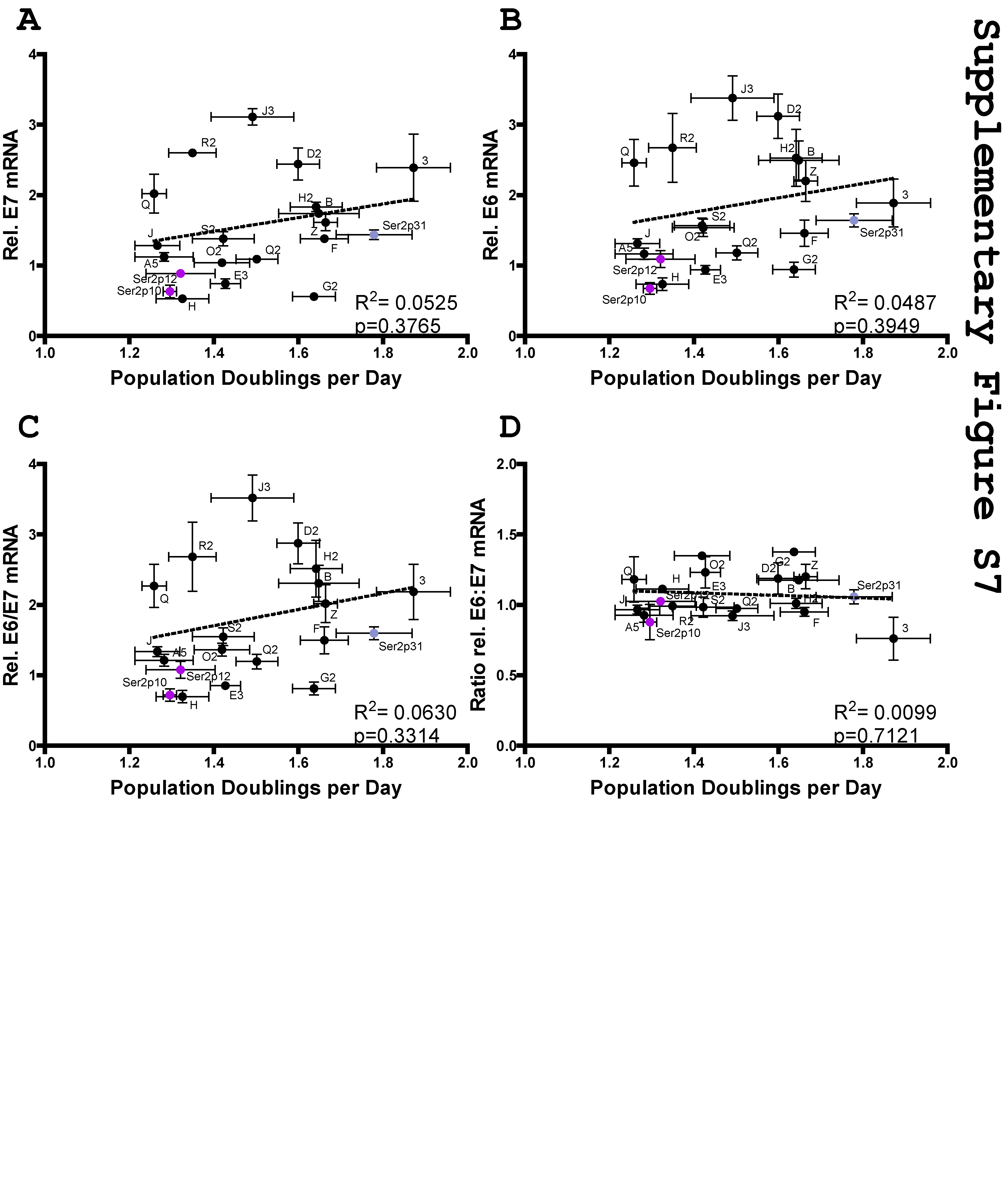

Supplement: Figure S7 — Relationships between cell growth rates and HPV16 transcript levels. [file path0233-0281-SD8.tif]
