# Supplementary material for: Virus transcript levels and cell growth rates after naturally occurring HPV16 integration events in basal cervical keratinocytes
Source: J Pathol. 2014 May 21;233(3):281–93. doi: 10.1002/path.4358 (PMC4285939; doi:10.1002/path.4358)
Supplement: Table S1 — Summary of the W12 cells studied. [file path0233-0281-SD10.doc]

**Supplementary Table S1. Summary of the W12 cells studied. All virus gene copy numbers were adjusted for cell ploidy (using mean values in the case of Q2 and 3) and rounded to the nearest whole number**

| **Cell line** | **Integration site** | **Ploidy** | **Copy number** | | | | |
| --- | --- | --- | --- | --- | --- | --- | --- |
|  |  |  | **E6** | **E7** | **Mean** **E6/E7** | **E2-5’** | **E2-3’** |
| **Clone Q2** | 1q44 | 2N / 4N | 8 | 10 | 9 | 8 | 6 |
| **Clone 3** | 2p24.1 | 2N / 3N | 14 | 16 | 15 | 1 | 1 |
| **Clone F** | 4q13.3 | 2N | 1 | 1 | 1 | 1 | 1 |
| **Clone H** | 4q21.23 | 2N | 1 | 1 | 1 | 0 | 1 |
| **Clone J** | 4q35.2 | 2N | 13 | 14 | 14 | 1 | 1 |
|  | 4q35.2 |  |  |  |  |  |  |
| **Clone Z** | 5q11.2 | 2N | 4 | 5 | 4 | 1 | 2 |
| **Clone A5** | 8p11.21 | 2N | 1 | 1 | 1 | 1 | 1 |
| **Clone B** | 8q24.21 | 2N | 3 | 4 | 4 | 1 | 1 |
|  | 8q24.21 |  |  |  |  |  |  |
| **Clone E3** | 9p24.3 | 4N | 8 | 9 | 9 | 9 | 7 |
| **Clone R2** | 10q22.1 | 2N | 6 | 6 | 6 | 0 | 5 |
| **Clone Q** | 12q14.3 | 4N | 5 | 6 | 6 | 1 | 1 |
|  | 8q24.21 |  |  |  |  |  |  |
| **Clone H2** | 17q12 | 2N | 7 | 8 | 8 | 6 | 6 |
|  | 17q12 |  |  |  |  |  |  |
| **Clone D2** | 18q21.2 | 2N | 3 | 4 | 4 | 0 | 3 |
| **Clone O2** | 19q13.31 | 2N | 2 | 2 | 2 | 1 | 3 |
| **Clone G2** | 21q22.1 | 2N | 3 | 3 | 3 | 3 | 0 |
| **Clone S2** | 22q12.1 | 2N | 6 | 6 | 6 | 6 | 5 |
| **Clone J3** | 8q24.21 | 3N | 5 | 6 | 5 | 1 | 1 |
| **W12Ser2 p31** | 8q24.21 | 2N | 3 | 5 | 4 | 4 | 4 |
| **W12Ser2 p10** | N/A | 2N | 140 | 120 | 130 | 158 | 141 |
| **W12Ser2 p12** | N/A | 2N | 150 | 150 | 150 | 150 | 150 |
