# Supplementary material for: Virus transcript levels and cell growth rates after naturally occurring HPV16 integration events in basal cervical keratinocytes
Source: J Pathol. 2014 May 21;233(3):281–93. doi: 10.1002/path.4358 (PMC4285939; doi:10.1002/path.4358)
Supplement: Table S2 — Primers and conditions for qPCR of HPV16 and cellular gDNA. [file path0233-0281-SD11.doc]

**Supplementary Table S2. Primers and conditions for qPCR of HPV16 and cellular gDNA**

|  | **Forward Primer (5’ to 3’)** | **Reverse Primer (5’ to 3’)** | **Reference/Supplier** |
| --- | --- | --- | --- |
| **E7** | AGGAGGATGAAATAGATGGTCCAG | CTTTGTACGCACAACCGAAGC | [18] |
| **E6** | GAGAACTGCAATGTTTCAGGACC | TGTATAGTTGTTTGCAGCTCTGTGC | [17] |
| **E2 5’** | GGAGACTCTTTGCCAACGTTTA | CACATTCTAGGCGCATGTGT | [32] |
| **E2 3’** | CTACATGGCATTGGACAGGA | GGTCACGTTGCCATTCACTA | [18] |
| **TLR2** | GGCCAGCAAATTACCTGTGTG | AGGCGGACATCCTGAACCT | [19] |
| **IFNβ** | TTGAATGGGAGGCTTGAATACTG | AATGCGGCGTCCTCCTTCT | Personal communication* |

Conditions used: 95°C for 2min; 45 cycles of 95°C for 15sec, 58°C for 20sec, 72°C for 15sec, 76°C for 5sec and read; final extension 78°C for 8min; followed by melting curve analysis from 65°C to 90°C to confirm product specific amplification.

*Annett Schoenemeyer, GlaxoSmithKline, UK-Virology, Medicine Research Centre, Gunnels Wood Road, Stevenage, UK
