# Supplementary material for: Virus transcript levels and cell growth rates after naturally occurring HPV16 integration events in basal cervical keratinocytes
Source: J Pathol. 2014 May 21;233(3):281–93. doi: 10.1002/path.4358 (PMC4285939; doi:10.1002/path.4358)
Supplement: Table S3 — Primers and conditions for qRT-PCR of HPV16 and housekeeping transcripts. [file path0233-0281-SD12.doc]

**Supplementary Table S3. Primers and conditions for qRT-PCR of HPV16 and housekeeping transcripts**

|  | **Forward Primer (5’ to 3’)^†^** | **Reverse Primer (5’ to 3’)^†^** | **Reference/Supplier** |
| --- | --- | --- | --- |
| **E7** | AGGAGGATGAAATAGATGGTCCAG | CTTTGTACGCACAACCGAAGC | [18] |
| **E6 all** | TGTTTCAGGACCCACAGGAGC | CGCAGTAACTGTTGCTTGCAG | [17] |
| **E6 full** | AGCGACCCAGAAAGTTACCA | GCATAAATCCCGAAAAGCAA | [18] |
| **E6*I** | AGGAGCGACCCAGAAAGTTA | TACGTGTTCTTATGATCTCACGTC | [31] |
| **E6*II** | AGGAGCGACCCAGAAAGTTA | TACGTGTTCTTATGATCTCACGTC | [31] |
| **E6*III** | AGGAGCGACCCAGAAAGTTA | GGATACTTCGTTGCTGCTCAC | Designed in-house |
| **E6*IV** | AGGAGCGACCCAGAAAGTTA | CTGGACCACGTCCTCACGT | Designed in-house |
| **E6*X** | AGGAGCGACCCAGAAAGTTA | AGTCACACTCACGTCGCA | Designed in-house |
| **E2 5’** | GGAGACTCTTTGCCAACGTTTA | CACATTCTAGGCGCATGTGT | [32] |
| **E2 3’** | CTACATGGCATTGGACAGGA | GGTCACGTTGCCATTCACTA | [18] |
| **GAPDH** | TGCACCACCAACTGCTTAGC | GGCATGGACTGTGGTCATGAG | [47] |
| **YWHAZ** | ACTTTTGGTACATTGTGGCTTCAA | CCGCCAGGACAAACCAGTAT | [47] |
| **RPL13A** | NA | NA | Qiagen (Hs_RPL13A_1_SG) |
| **TBP** | NA | NA | Qiagen (Hs_TBP_1_SG) |

Conditions used: 95°C for 2min; 45 cycles of 95°C for 15sec, 58°C for 20sec, 72°C for 15sec, 76°C for 5sec and read; final extension 78°C for 8min; followed by melting curve analysis from 65°C to 90°C to confirm product specific amplification.

†NA: Sequence information not available.
