# Supplementary material for: Virus transcript levels and cell growth rates after naturally occurring HPV16 integration events in basal cervical keratinocytes
Source: J Pathol. 2014 May 21;233(3):281–93. doi: 10.1002/path.4358 (PMC4285939; doi:10.1002/path.4358)
Supplement: Table S4 — Primers and conditions for ChIP–qPCR of HPV16 and cellular chromatin. [file path0233-0281-SD13.doc]

Supplementary Table S4. Primers and conditions for ChIP–qPCR of HPV16 and cellular chromatin

|  | **Forward Primer (5’ to 3’)** | **Reverse Primer (5’ to 3’)** | **Reference/Supplier** |
| --- | --- | --- | --- |
| **HPV16**  **7419F to 7552R** | TTTGTAGCGCCAGCGGCCATTT | GCATGGCAAGCAGGAAACGTACAA | Designed in-house |
| **HPV16**  **7854F to 65R** | GCAAACCGTTTTGGGTTACA | ACTAACCGGTTTCGGTTCAA | [19] |
| **HPV16**  **111F to 223R** | AGGACCCACAGGAGCGACCC | ACGTCGCAGTAACTGTTGCTTGCA | Designed in-house |
| **HPV16**  **427F to 506R** | GCCACTGTGTCCTGAAGAAAAGCA | GACCGGTCCACCGACCCCTT | Designed in-house |
| **HPV16**  **649F to 765R** | GACAGCTCAGAGGAGGAGGA | GCACAACCGAAGCGTAGAGT | [19] |
| **HPV16**  **1250F to 1368R** | GCGAAGACAGCGGGTATGGCA | GCAACCACCCCCACTTCCACC | Designed in-house |
| **GAPDHprom** | CGGCTACTAGCGGTTTTACG | AAGAAGATGCGGCTGACTGT | [48] |
| **γ-Globinprom** | GCCTTGACCAATAGCCTTGACA | GAAATGACCCATGGCGTCTG | [48] |

Conditions used: 95°C for 2min; 45 cycles of 95°C for 15sec, 58°C for 20sec, 72°C for 15sec, 76°C for 5sec and read; final extension 78°C for 8min; followed by melting curve analysis from 65°C to 90°C to confirm product specific amplification.
