# Supplementary material for: Virus transcript levels and cell growth rates after naturally occurring HPV16 integration events in basal cervical keratinocytes
Source: J Pathol. 2014 May 21;233(3):281–93. doi: 10.1002/path.4358 (PMC4285939; doi:10.1002/path.4358)
Supplement: Supplementary file 13 — Appendix S1. Supplementary Information. [file path0233-0281-SD1.doc]

**SUPPORTING INFORMATION**

**Supplementary materials and methods**

***Calculation of population doubling times***

Cells were seeded at 5x10^4^ cells per well and quantified every 24 hours during log growth phase, using trypan blue staining and a Countess automated cell counter (Invitrogen). Cell numbers were determined from the mean of three replicate wells, using the mean of two counts for each well. Cell numbers were plotted on log_2_ transformed scale and the population doubling rate taken from the linear section of the curve, as described .

***Analysis of phenotype in organotypic tissue culture***

Collagen rafts incorporating mouse dermal papilla cells were prepared in 24-well plates, as described . The W12 clones were seeded onto each raft (1x10^5^ cells) and grown to confluency, after which the rafts were raised to an air-liquid interface for 9–12 days. Following formalin fixation, rafts were processed to paraffin and stained by hematoxylin and eosin (H&E). Immunohistochemistry (IHC) was performed as described , to detect the presence of the cell-cycle protein minichromosome maintenance protein 2 (MCM2; using ‘in house’ primary antibodies) and the squamous differentiation markers cytokeratins 10/13 (KRT10/13; DAKO) .

***Quantification of HPV16 gene copy number***

Copy numbers of the HPV16 early genes E7, E6 and E2 were determined using SYBR Green (Sigma-Aldrich) quantitative PCR (qPCR) of genomic DNA, as described . Copy number was calculated using the Pfaffl equation , relative to the Ct values generated for the control genes *IFNβ* and *TLR2*. The latter were previously shown not to undergo copy number change in multiple W12 populations, including over long-term culture . qPCR was carried out using an Eppendorf Mastercycler epgradient-S. All primers and conditions used are described in Supplementary Table 2. Copy number values were adjusted for the ploidy of each cell line, determined by propidium iodide staining and flow cytometry, with reference to the control haploid cell line CAL51 .

***Quantification of HPV16 transcripts***

Total RNA was harvested from cells *in situ* using TRIzol (Invitrogen) and cDNA prepared using Quantitect Reverse Transcription (Qiagen). Transcript levels per cell of HPV16 early genes E7, E6 and E2 were determined using SYBR Green quantitative reverse transcription-PCR (qRT-PCR). In this, the E7 primers detected all transcripts encoding the E7 protein. We used a range of primer pairs to quantify total E6 transcripts, as well as the alternatively spliced forms, E6*I, E6*II, E6*III E6*IV, E6*X and full length E6 . Separate primer pairs were used to quantify the 5’ and 3’ ends of E2 . The qRT-PCR was carried out using an Eppendorf Mastercycler epgradient-S. All primers and conditions used are described in Supplementary Table 3.

Relative transcript expression levels were determined using the Pfaffl equation . Values were normalised to the Ct values generated for control house-keeping genes *GAPDH*, *RPL13A*, *TBP* and *YWHAZ*, using the mean of the four comparisons in each case . All values were referenced to those in an independent population of W12 cells containing ~150 episomes per cell, W12 Series-6 p11 (W12Ser6p11). In addition, we combined the HPV16 expression and gene copy number quantification data to determine the levels of transcription per DNA template copy for E7, E6 and E2, as described .

***Quantitative Western immunoblotting***

To measure levels of HPV16 E7 and E6 proteins, total protein extracts were harvested from cells *in situ* using RIPA buffer (Sigma-Aldrich) supplemented with protease and phosphatase inhibitor (Pierce). To quantify HPV16 E2 protein, nuclear protein extracts were harvested as described . Quantitative Western immunoblotting was carried out as described using primary antibodies against HPV16 E7 (sc-6981, Santa Cruz; 1:300), HPV16 E6 (1E-6F4, Euromedex; 1:8,000) and HPV16 E2 (TVG261, Cancer Research Technology; 1:2,500), with β-actin (ab6276, Abcam; 1:150,000) as the loading control. None of the antibodies against HPV proteins produced bands in HPV-negative NCx cells ([17](../../../../D:%5CUsers%5CCinzia%5CDocuments%5CHPV%20work%5CKelties%20clones%20work%5Cpaper%5CJournal%20of%20Pathology%20submission%5CScarpini%20et%20al%20Supplementary%20Information_v16.docx#_ENREF_17) and data not shown). All gels included a series of standards (W12 clone B total protein extracts, loaded from 10 to 100μg per lane), plus a reference sample (extracts of independent episome-containing W12Ser6p11 cells). Each test sample was loaded at 50μg total protein or 25μg nuclear protein per lane. For each antibody, standard curves were generated from the W12 clone B series and used to calculate the amount of target protein in each test sample, referenced to levels in the W12Ser6p11 sample.

***Chromatin immunoprecipitation (ChIP)***

ChIP and subsequent qPCR were performed as described . The ChIP-validated primary antibodies used (all Active Motif) were against: histone H3 (H3; 39163), acetylated histone H3 (H3ac; 39139), trimethylated lysine 4 of histone H3 (H3K4me3; 39915), dimethylated lysine 9 of histone H3 (H3K9me2; 39239) and dimethylated lysine 27 of histone H3 (H3K27me2; 39245). Pre-immune rabbit serum (X0902, Dako) was used as a negative control for background signal. PCR quantification of HPV16 DNA sequences in the immunoprecipitated chromatin covered six sites along the HPV16 genome, from the long control region to the E1 open reading frame (Supplementary Table 4). Fold enrichment levels for each histone modification were referenced to background H3 levels at each site. Amplification of the GAPDH and γ-globin promoters was used to control for the efficiency of immunoprecipitation of active and repressed histone modifications, respectively .

**References**

**(Note: reference numbers correspond to reference list in main article)**

17. Herdman MT, Pett MR, Roberts I, *et al.* Interferon-beta treatment of cervical keratinocytes naturally infected with human papillomavirus 16 episomes promotes rapid reduction in episome numbers and emergence of latent integrants. *Carcinogenesis* 2006; **27**: 2341-2353.

19. Gray E, Pett MR, Ward D, *et al.* *In vitro* progression of human papillomavirus 16 episome-associated cervical neoplasia displays fundamental similarities to integrant-associated carcinogenesis. *Cancer Res* 2010; **70**: 4081-4091.

22. Pett MR, Alazawi WO, Roberts I, *et al.* Acquisition of high-level chromosomal instability is associated with integration of human papillomavirus type 16 in cervical keratinocytes. *Cancer Res* 2004; **64**: 1359-1368.

29. Coleman N, Greenfield IM, Hare J, *et al.* Characterization and functional analysis of the expression of intercellular adhesion molecule-1 in human papillomavirus-related disease of cervical keratinocytes. *Am J Pathol* 1993; **143**: 355-367.

31. Cricca M, Venturoli S, Leo E, *et al.* Molecular analysis of HPV 16 E6I/E6II spliced mRNAs and correlation with the viral physical state and the grade of the cervical lesion. *J Med Virol* 2009; **81**: 1276-1282.

32. Hanning JE, Groves IJ, Pett MR, *et al.* Depletion of polycistronic transcripts using short interfering RNAs: cDNA synthesis method affects levels of non-targeted genes determined by quantitative PCR. *Virol J* 2013; **10**: 159.

43. Gonzalez MA, Pinder SE, Callagy G, *et al.* Minichromosome maintenance protein 2 is a strong independent prognostic marker in breast cancer. *J Clin Oncol* 2003; **21**: 4306-4313.

44. Smedts F, Ramaekers FC, Vooijs PG. The dynamics of keratin expression in malignant transformation of cervical epithelium: a review. *Obstet Gynecol* 1993; **82**: 465.

45. Pfaffl MW. A new mathematical model for relative quantification in real-time RT-PCR. *Nucleic Acids Res* 2001; **29**: e45.

46. Gioanni J, Le Francois D, Zanghellini E, *et al.* Establishment and characterisation of a new tumorigenic cell line with a normal karyotype derived from a human breast adenocarcinoma. *Br J Cancer* 1990; **62**: 8-13.

47. Vandesompele J, De Preter K, Pattyn F, *et al.* Accurate normalization of real-time quantitative RT-PCR data by geometric averaging of multiple internal control genes. *Genome Biol* 2002; **3**: RESEARCH0034.

48. Groves IJ, Reeves MB, Sinclair JH. Lytic infection of permissive cells with human cytomegalovirus is regulated by an intrinsic ‘pre-immediate-early’ repression of viral gene expression mediated by histone post-translational modification. *J Gen Virol* 2009; **90**: 2364-2374.

**Legends to Supplementary Figures and Tables**

**Supplementary Figure 1. Expression of alternative E6 transcripts.** The graphs show levels of the alternative E6 transcripts, E6 full length, E6*I, E6*II, E6*III, E6*IV and E6*X, plotted against total levels of E7 mRNA (left column) and E6 mRNA (right column). The correlation data are for the 18 clones (black circles). Results for the W12Ser2p31 cells (pale blue circles) and W12Ser2p10 and W12Ser2p12 cells (purple circles) are also shown. Error bars = SEM. Rel. = Relative.

**Supplementary Figure 2. Relationship between DNA copy number for HPV16 E2-5' and E2-3'.** The correlation data are for the 18 clones (black circles). Results for the W12Ser2p31 cells (pale blue circles) and W12Ser2p10 and W12Ser2p12 cells (purple circles) are also shown. Error bars = SEM.

**Supplementary Figure 3. Relationships between expression levels and DNA copy number for HPV16 E7 (A), E6 (B), and E2-5' (C).** E6 expression refers to total levels of E6 transcripts. The correlation data are for the 18 clones (black circles). Results for the W12Ser2p31 cells (pale blue circles) and W12Ser2p10 and W12Ser2p12 cells (purple circles) are also shown. Error bars = SEM. Rel. = Relative.

**Supplementary Figure 4. Expression levels per template of HPV16 E2-5' and E2-3' mRNA.** Samples are ordered according to expression levels of E7 mRNA per template (as shown in Figure 3A). The 18 W12 clones are shown by orange bars, the W12Ser2p31 cells by pale blue bars and the W12Ser2p10 and W12Ser2p12 cells by purple bars. In all cases, open bars refer to E2-5', while hatched bars refer to E2-3'. Error bars = SEM.

**Supplementary Figure 5. Western blot analysis of HPV16 E2 protein levels in representative samples.** The panel shows a western blot for E2 protein expression in representative W12 clones (A5, B, G2, Q2, S2), plus the positive control cells W12Ser2p15 and the negative control cells NCx/2 (normal ectocervical primary cultures), HaCaT (near-normal epidermal keratinocytes) and HeLa (HPV18-positive cervical adenocarcinoma cells).

**Supplementary Figure 6. Relationships between levels of HPV16 oncoproteins and alternative E6 transcripts.** The graphs plot levels E6 protein (left column) or E7 protein (right column) vs. levels of the alternative E6 transcripts, E6 full length, E6*I, E6*II, E6*III, E6*IV and E6*X. The correlation data are for the 18 clones (black circles). Results for the W12Ser2p31 cells (pale blue circles) and W12Ser2p10 and W12Ser2p12 cells (purple circles) are also shown. Error bars = SEM. Rel. = Relative. A.U. = Arbitrary Units.

**Supplementary Figure 7. Relationships between cell growth rates and HPV16 transcript levels.** The graphs plot growth rates vs. transcript levels for E7 (A), E6 (B), the mean E6/E7 values (C) and the E7:E6 ratios (D). The correlation data are for the 18 clones (black circles). Results for the W12Ser2p31 cells (pale blue circles) and W12Ser2p10 and W12Ser2p12 cells (purple circles) are also shown. Error bars = SEM. Rel. = Relative.

**Supplementary Figure 8. Relationships between cell growth and alternative E6 transcripts.** The graphs plot cell growth vs. levels of the alternative E6 transcripts, E6 full length, E6*I, E6*II, E6*III, E6*IV and E6*X. The left hand column shows data for the E6 alternative transcripts in isolation, while the right hand column shows data for the mean levels of the E6 alternative transcripts and E7. The correlation data are for the 18 clones (black circles). Results for the W12Ser2p31 cells (pale blue circles) and W12Ser2p10 and W12Ser2p12 cells (purple circles) are also shown. Error bars = SEM. Rel. = Relative.

**Supplementary Table 1. Summary of the W12 cells studied.** All virus gene copy numbers were adjusted for cell ploidy (using mean values in the case of Q2 and 3) and rounded to the nearest whole number.

**Supplementary Table 2. Primers and conditions for qPCR of HPV16 and cellular gDNA.**

**Supplementary Table 3. Primers and conditions for qRT-PCR of HPV16 and housekeeping transcripts.**

**Supplementary Table 4. Primers and conditions for ChIP–qPCR of HPV16 and cellular chromatin.**
